# Jaw-in-a-Riley-Day: Mandibular Free Flap Reconstruction With Virtual Surgical Planning in a Patient With Familial Dysautonomia

**DOI:** 10.7759/cureus.26336

**Published:** 2022-06-26

**Authors:** Kyohei Itamura, Steven Kupferman, Jay Lee, Jon Mallen-St. Clair

**Affiliations:** 1 Otolaryngology - Head and Neck Surgery, Cedars-Sinai Medical Center, Los Angeles, USA; 2 Oral and Maxillofacial Surgery, Cedars-Sinai Medical Center, Los Angeles, USA

**Keywords:** free fibula flap, digital planning, mandibular trauma, microvascular surgery, mandibular reconstruction

## Abstract

We present a unique case of mandibular reconstruction using virtual surgical planning (VSP) of a post-traumatic mandibular non-union defect for a patient with familial dysautonomia (FD), also known as Riley-Day Syndrome. In this case, the complexity related to perioperative and surgical challenges illustrates the utility of VSP and the importance of multi-disciplinary collaboration in jaw-free flap reconstructive surgery. We highlight our experience with the “Jaw-In-A-Day” approach in conjunction with tailored preoperative planning and perioperative care resulting in successful mandibular free flap reconstruction.

## Introduction

The “Jaw-In-A-Day” (JIAD) approach for the reconstruction of mandibular and maxillofacial defects has become a well-recognized technique to accomplish resection, reconstruction, and dental rehabilitation in one operation [1]. This approach, pioneered in 2013 by Levine et al., relies on patient-specific virtual preoperative planning as well as multi-disciplinary collaboration to maximize operative efficiency [2].

With modern advances in digital technology, there has been a progressive expansion in reconstructive capabilities through JIAD. Virtual surgical planning (VSP) has increased the range of surgical candidates as more complex defects are able to be accurately reconstructed. Thus far in the literature, JIAD has been well-described for reconstruction and immediate dental rehabilitation after resection of mandibular or maxillary tumors such as ameloblastoma [3].

We present the first reported case in the literature of JIAD being applied to a patient with a rare genetic syndrome. Familial Dysautonomia (FD), also known as Riley-Day Syndrome, is a rare autosomal recessive disease that predominantly affects the Ashkenazi Jewish population due to a pathogenic variant of the ELP1 gene. It is characterized by a developmental defect of sympathetic, parasympathetic, and sensory neurons, leading to multi-organ autonomic and sensory dysfunction. Patients present with a variety of symptoms related to dysautonomia, including labile hemodynamics, gastrointestinal dysmotility, oropharyngeal dysphagia, and altered pain and temperature sensation [4]. In response to stressful stimuli including emotional or physical stressors, patients can acutely experience an autonomic crisis characterized by hypertension, tachycardia, breathing irregularities, nausea, retching, and vomiting. Although maxillofacial deformity is not a classic finding associated with FD, significant defects can be acquired secondarily through trauma due to a higher propensity for skeletal fractures [3]. 

The following case posed both peri- and intraoperative challenges that to our knowledge have not been described in the JIAD literature. We discuss VSP and multi-disciplinary collaboration as keystones for successful jaw reconstructive surgery, especially in this medically complex patient.

## Case presentation

**Figure 1:**
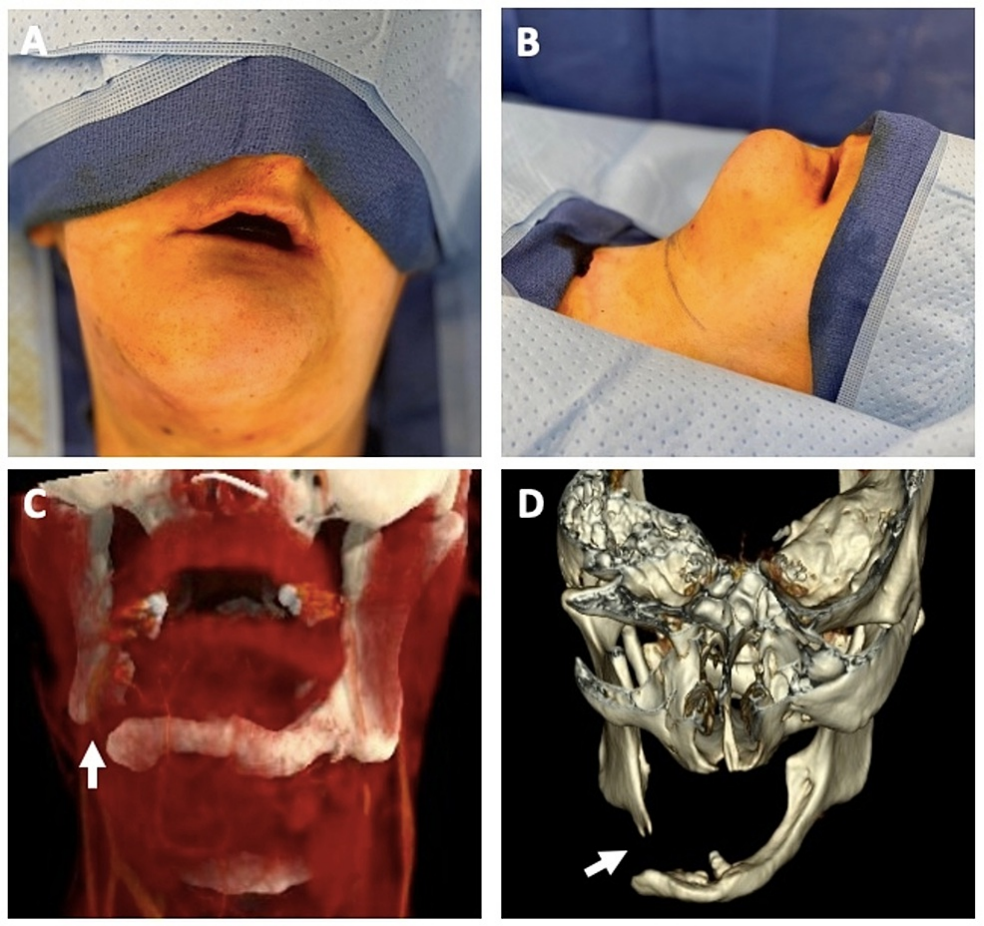
Preoperative mandibular non-union defect Preoperative mandibular non-union defect in frontal view (A), left lateral view (B), CT 3-dimensional reconstruction with soft tissue superimposition in frontal view (C), CT 3-dimensional reconstruction from superior view showing anterior displacement of the left mandible segment. White arrows indicate non-union defect.

A 29-year-old female with FD presented to the emergency department with submental swelling. She has a history of facial trauma several years prior to a presentation that was left untreated. Since then, she has had a mandibular deformity complicated by recurrent episodes of mandibular osteomyelitis, submental cellulitis, and a draining orocutaneous fistula requiring multiple courses of intravenous antibiotic treatment. On the initial exam, there was a palpable right-sided mandibular defect with firm submental edema and overlying skin erythema. CT neck with contrast at that time demonstrated a right 1.5cm submental abscess and mandibular non-union defect with extensive destructive changes in the symphysis and body (Figures 1C, 1D). The abscess was drained under local anesthesia, and the patient was discharged with six weeks of intravenous antibiotics. The need for mandibular reconstruction was discussed extensively with the family. The goals of the operation were to avoid future recurrent infections as well as to improve oral function. The patient was deemed a candidate for surgery after the infection was treated with a full course of antibiotics. An immediate preoperative exam was significant for a completely edentulous mandible and partially edentulous maxilla with obvious mandibular disfigurement and chin asymmetry (Figures 1A, 1B).

Pre-operative planning

**Figure 2:**
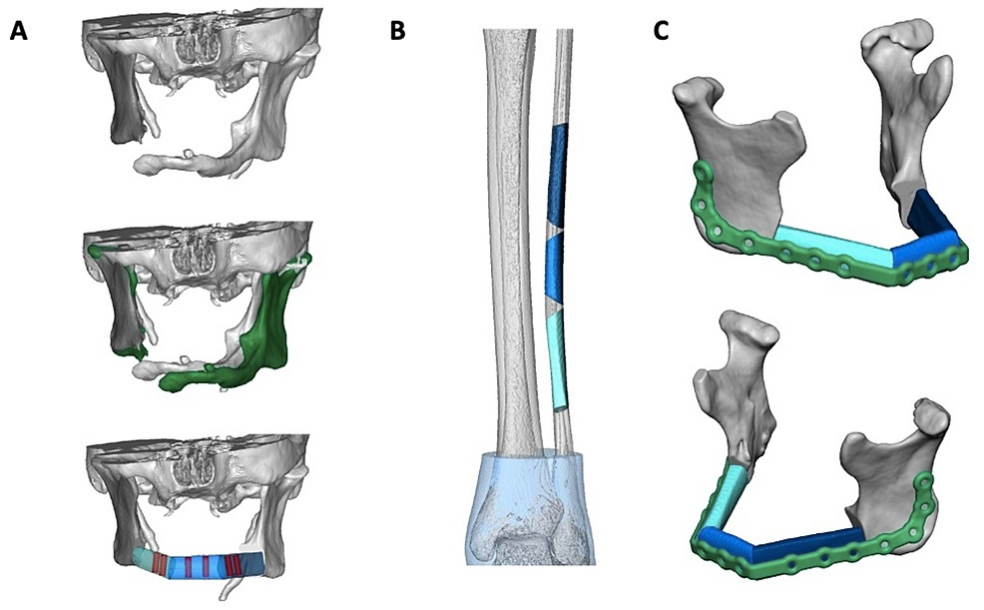
Preoperative virtual surgical planning Preoperative virtual surgical planning schematics for mandibular resection (A), left fibula donor site (B), and plating of fibular free flap on the mandible (C).

Given the patient’s significant past medical history of FD, several operative challenges were considered, especially relating to perioperative care. These risks were discussed with the patient and family. Of note, the patient previously had several surgeries requiring general anesthesia, which she tolerated without major complications. Informed consent was obtained after the decision was made to proceed with JIAD surgery, including angle-to-angle mandibular resection followed by fibula free flap (FFF) reconstruction with simultaneous dental implantation, by the oral maxillofacial surgery (OMFS) and otolaryngology (ENT) teams. Due to the complexity of the case, virtual surgical planning (VSP) was employed (Figures 2A-2C). The extent of resection of non-viable mandible was determined and cutting and drilling guides were prefabricated according to VSP schematics in preparation for surgery.

Surgical procedure

After preprocedural anxiolysis with midazolam, successful induction of anesthesia with propofol bolus and neuromuscular paralysis with rocuronium was achieved for oral intubation with a size 6-0 endotracheal tube. Tracheostomy was then performed by the OMFS team followed by angle-to-angle mandibular resection and preparation of the resection bed. Once the airway was secured via tracheostomy, an incision was made in an existing neck crease from the left to right mandibular angles. Dissection was performed down to the left pterygomandibular sling to expose the left mandible from the angle to midline. The right pterygomasseteric sling was then exposed and the right mandible was visualized, including the non-union defect. Significant soft tissue scarring was noted in the defect area. Bilateral slings were excised and the mandible was skeletonized. Cutting and drilling guides prefabricated with the VSP system were secured and osteotomies were performed to resect the mandible from angle to angle. Prediction holes on the guides were drilled in preparation for inset of the FFF later.

**Figure 3:**
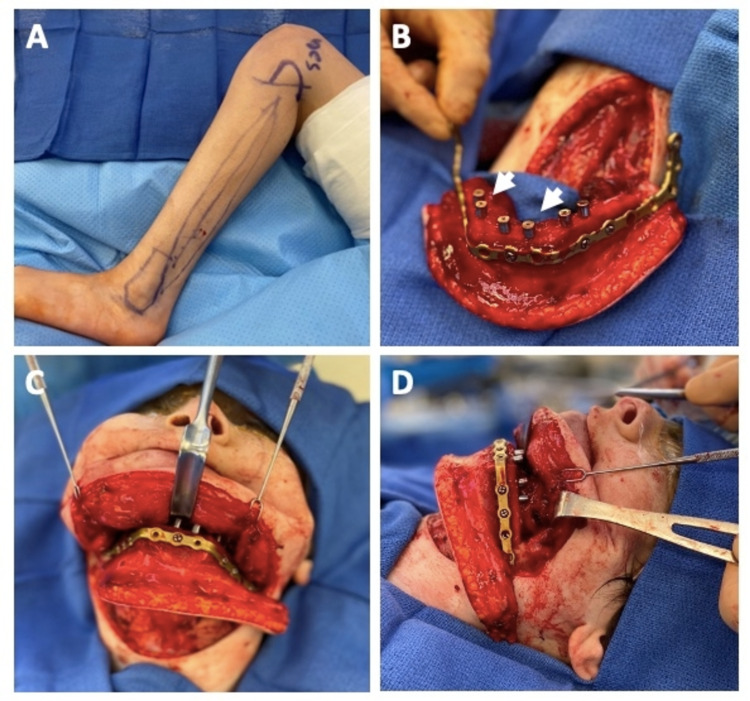
Intraoperative free flap harvest and inset Intraoperative photos of incision markings for the left fibular free flap donor site (A), placement of dental implants in and pre-plating of the osseocutaneous fibular free flap prior to flap harvest (B), flap inset through neck incision from a frontal (C), and left lateral view (D). White arrows indicate dental implant abutments in the fibular free flap.

Concurrently, the left FFF was harvested by ENT (Figure 3A). Dissection was carried down to the fibula in the standard fashion with protection of neurovascular structures. The fibula was released with inferior and superior cuts using a sagittal saw with preservation of at least 6 cm of bone toward the ankle and knee joints. Using prefabricated cutting guides, two closing wedge osteotomies were made. The prefabricated reconstruction plate was then fixated to the fibula using 6 mm 2.0 locking screws. Six individual 3.75 mm x 10 mm narrow platform parallel conical connection dental implants were placed into pre-planned locations on the fibula using a standard drilling protocol by OMFS. Six individual 7 mm x 5 mm healing abutments were then placed onto the dental implants with excellent stability (Figure 3B).

**Figure 4:**
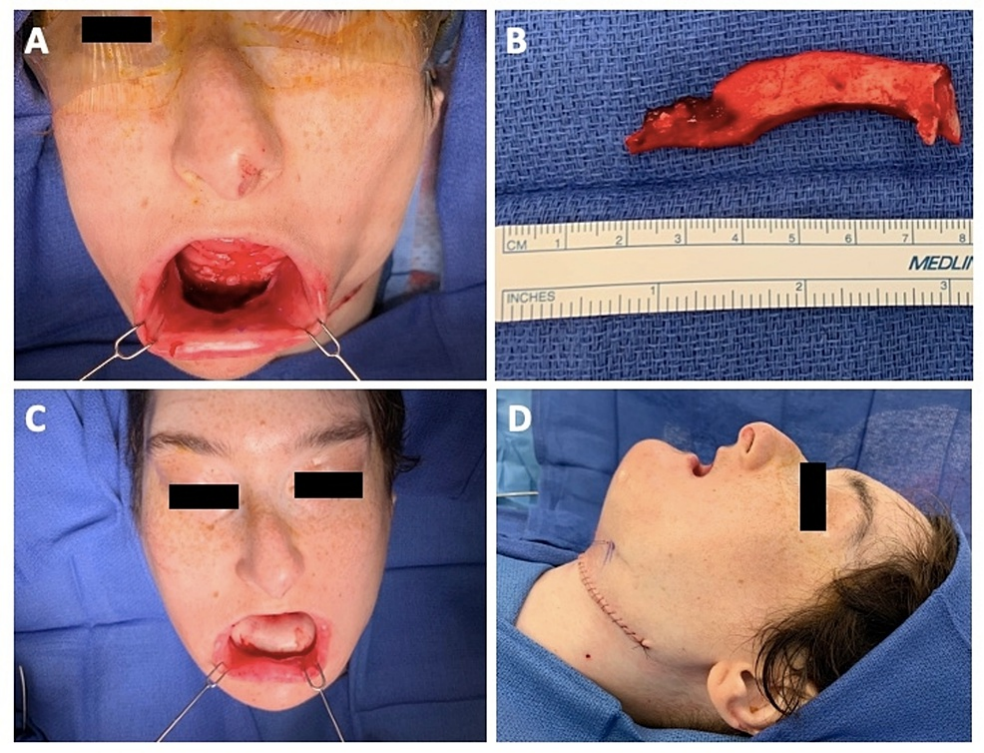
Mandibular defect specimen and patient at conclusion of case Immediately postoperative photos showing successful inset of fibular free flap (A), resected segment of mandibular defect (B), inset of fibular free flap skin paddle, left lateral view with primary closure of the neck (D).

The FFF with the vascular pedicle was then harvested with a 3-cm wide skin paddle and transferred to the resection bed where it was inset and then secured with locking screws at the predrilled prediction holes (Figures 3C, 3D). The donor peroneal artery and vein were anastomosed to the recipient's left facial artery and vein, respectively, using microvascular technique. The operation proceeded to completion with placement of perforated drains in the neck and the leg followed by closure of fascia and skin (Figures 4A-4D).

From an anesthesia standpoint, the patient was sedated on a continuous infusion of propofol at 30-50 mcg/kg/min and fentanyl 1-1.5 mcg/kg/hr. Hypotensive episodes were controlled with both intravenous fluid administration of plasmalyte and 5% albumin solutions. For additional pressure support, phenylephrine drip from 5 to 15 mcg/min was used to titrate to a goal systolic blood pressure of 100-120 mmHg. No blood transfusions were required. There were no noted anesthetic complications during the procedure. The patient was transferred to the surgical intensive care unit (SICU) for hemodynamic monitoring, ventilatory support, and frequent flap checks after completing the case.

Postoperative course

SICU course was remarkable for labile blood pressures and persistent nausea. On postoperative day (POD) 1, one unit of packed red blood cells was transfused for a hemoglobin of 6.7 g/dL. On POD 2, the patient had an autonomic crisis episode characterized by hypertension, nausea, retching, and tachycardia, which was controlled with dexmedetomidine drip. She was eventually weaned off ventilatory support and vasoactive drips and was subsequently transferred to the general medical-surgical unit on POD 5 where she continued to improve clinically. Nausea episodes were controlled with as needed doses of metoclopramide. Blood pressure remained transiently labile with intermittent asymptomatic hypotensive episodes, which were responsive to intravenous fluid boluses. Flap health was closely monitored throughout the patient’s hospital stay with no loss of doppler signal or concerning changes on exam. Tracheostomy tube was successfully downsized and eventually decannulated. She was advanced progressively to a soft diet by mouth from nasogastric tube feeding. The patient was discharged to home on POD 14 without complications.

**Figure 5:**
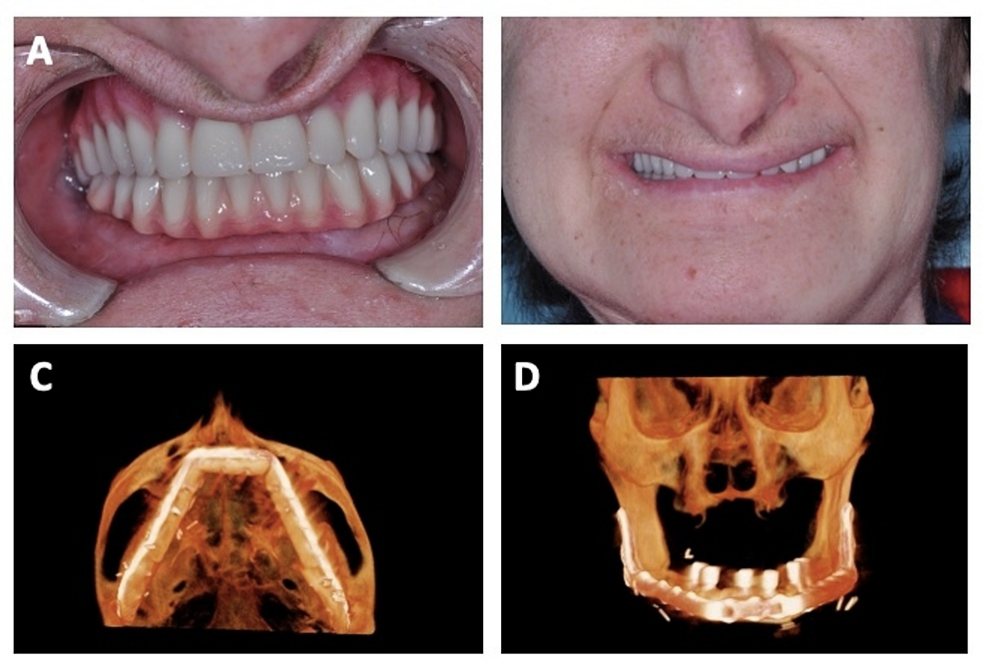
Six months postoperative follow-up with placement of crown prostheses Six-month postoperative photos after placement of crown prostheses into intraoperatively placed dental implants in close-up view showing grossly normal occlusion (A) and with patient smiling. Six-month postoperative CT three-dimensional reconstruction scan from bottom (C) and frontal views (D).

The patient continued to recover well during her post-hospital course. Crown prostheses were placed at postoperative month 6 into the dental implants (Figures 5A-5D). At the time of this case report, approximately 17 months after surgery, she was noted to have continued satisfaction with her aesthetic and functional ability to eat a regular diet. There were no reported complications related to her JIAD surgery.

## Discussion

Here we describe the first reported case in the literature of a patient with FD, also known as Riley-Day Syndrome, undergoing JIAD free flap reconstructive surgery for a post-traumatic mandibular non-union defect. Surgical care of FD patients poses a considerable challenge. Hemodynamic lability, central sleep apnea, electrolyte imbalances, and autonomic crisis are prominent concerns that must be planned for, among many others [4]. Free flap microvascular surgery of any kind for these patients is yet to be reported in the literature. Theoretically, large hemodynamic shifts due to dysautonomia can be a significant risk to free flap viability in the acute postoperative setting if not controlled [5]. A direct alpha-1 adrenergic agonist, in our case continuous phenylephrine infusion, was used perioperatively to correct hypotension [6]. Intravenous fluids were judiciously administered in small boluses to avoid fluid overload [7]. Dexmedetomidine infusion as reported in the literature proved useful for sequestering the autonomic crisis episode encountered in our patient [8].

With an improved understanding of the mechanism of FD and better titration of medications and measures to control autonomic episodes, a variety of complex surgical cases have been reported without significant morbidity or mortality [4]. Even still, minimizing exposure to multiple anesthetic and surgical events remains prudent in these patients. Our patient, therefore, was a candidate for JIAD wherein her mandibular defect, causing the significant quality of life issues, could be addressed all in one surgery. The key to the case presented was the multidisciplinary team consisting of OMFS, ENT, anesthesiology, intensive care, internal medicine, and nursing care who all understood and implemented measures to mitigate the risk of autonomic crisis to help the patient recover through the acute postoperative period while maintaining flap health.

A recent case series by Khatib et al. discussed the increased reliability of JIAD as well as the improved availability of digital tools since its inception within the past decade [9]. Most cases thus far in the literature have entailed reconstruction after resection of maxillary or mandibular tumors, mainly ameloblastoma. VSP has become a highly applicable tool for these large maxillofacial tumors and the reconstruction of complex traumatic defects and osteoradionecrosis of the jaw. [10]. Our group recently applied JIAD to a patient with bisphosphonate-related osteonecrosis of the jaw, thus expanding the variety of mandibular pathologies that can possibly benefit from this surgery [11]. However, more investigation is required to elucidate long-term outcomes and the impact of JIAD surgery on patient quality of life.

## Conclusions

We present the JIAD surgical approach for mandibular reconstruction applied to a medically complex patient with a rare genetic condition called FD, also known as Riley-Day Syndrome. Virtual surgical planning and multi-disciplinary collaboration were keys to caring for this case. Although more investigation is necessary to quantify the long-term outcomes of JIAD, these fundamental concepts provide a basis for applying this evolving surgical approach to other unique patients.
